# Targeted serum proteomics of longitudinal samples from newly diagnosed youth with type 1 diabetes affirms markers of disease

**DOI:** 10.1007/s00125-025-06394-7

**Published:** 2025-02-28

**Authors:** Robert Moulder, M. Karoliina Hirvonen, Tommi Välikangas, Tomi Suomi, Lut Overbergh, Mark Peakman, Søren Brunak, Chantal Mathieu, Mikael Knip, Laura L. Elo, Riitta Lahesmaa, on behalf of the INNODIA consortium

**Affiliations:** 1 Turku Bioscience Centre, University of Turku and Åbo Akademi University, Turku, Finland; 2 InFLAMES Research Flagship Centre, University of Turku, Turku, Finland; 3 Katholieke Universiteit Leuven/Universitaire Ziekenhuizen, Leuven, Belgium; 4 Immunology & Inflammation Research Therapeutic Area, Sanofi, Cambridge, UK; 5 Novo Nordisk Foundation Center for Protein Research, Faculty of Health and Medical Sciences, University of Copenhagen, Copenhagen, Denmark; 6 Research Program for Clinical and Molecular Metabolism, Faculty of Medicine, University of Helsinki, Helsinki, Finland; 7 Pediatric Research Centre, New Children’s Hospital, Helsinki University Hospital, Helsinki, Finland; 8 Department of Pediatrics, Tampere University Hospital, Tampere, Finland; 9 Institute of Biomedicine, University of Turku, Turku, Finland

**Keywords:** C-peptide, Markers, Serum proteomics, Targeted proteomics, Type 1 diabetes

## Abstract

**Aims/hypothesis:**

While investigating markers for declining beta cell function in type 1 diabetes, we previously demonstrated 11 statistically significant protein associations with fasting C-peptide/glucose ratios in longitudinal serum samples from newly diagnosed (ND) individuals (*n*=86; 228 samples in total) participating in the INNODIA (Innovative approaches to understanding and arresting type 1 diabetes) study. Furthermore, comparison with protein measurements from age- and sex-matched autoantibody-negative unaffected family members (UFMs, *n*=194) revealed differences in the serum levels of 13 target proteins. To further evaluate these findings, we analysed longitudinal serum drawn during the first year after diagnosis from a new group of ND individuals subsequently enrolled in the study, together with samples from additional UFMs.

**Methods:**

To validate the previously reported statistically significant protein associations with type 1 diabetes progression, selected reaction monitoring (SRM) MS analyses were carried out. Sera from individuals diagnosed with type 1 diabetes under the age of 18 years (*n*=146) were collected within 6 weeks of diagnosis and at 3, 6 and 12 months after diagnosis (560 samples in total). The resulting SRM data were compared with fasting C-peptide/glucose measurements, which were used as a proxy for beta cell function. The protein data were further compared with cross-sectional SRM measurements from age- and sex-matched UFMs (*n*=272).

**Results:**

Our results confirmed the presence of significant (*p*<0.05) inverse associations between fasting C-peptide/glucose ratios and peptides from apolipoprotein B-100, apolipoprotein M and glutathione peroxidase 3 (GPX3) in ND individuals. Additionally, we observed consistent differences in the levels of ten of the 13 targeted proteins between individuals with type 1 diabetes and UFMs. These proteins included GPX3, transthyretin, prothrombin, apolipoprotein C1 and afamin.

**Conclusions/interpretation:**

The validated results reflect the landscape of biological changes accompanying type 1 diabetes. For example, the association of the targeted apolipoproteins with fasting C-peptide/glucose ratios in the first year after diagnosis is likely to relate to lipid abnormalities observed in individuals with type 1 diabetes, and reiterates the connection of apolipoproteins with the underlying changes accompanying the disease. Further research is needed to explore the clinical value and relevance of these targets.

**Graphical Abstract:**

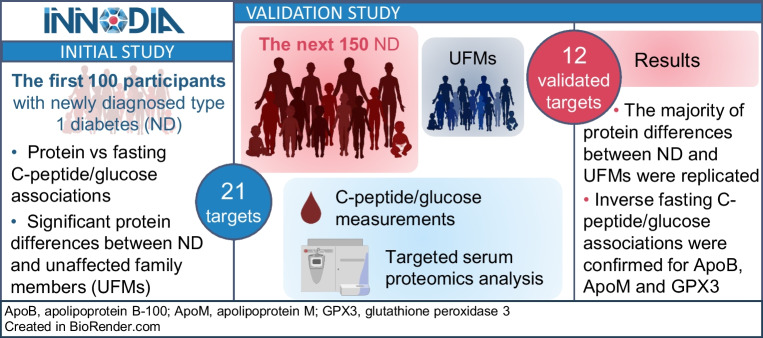

**Supplementary Information:**

The online version of this article (10.1007/s00125-025-06394-7) contains peer-reviewed but unedited supplementary material.


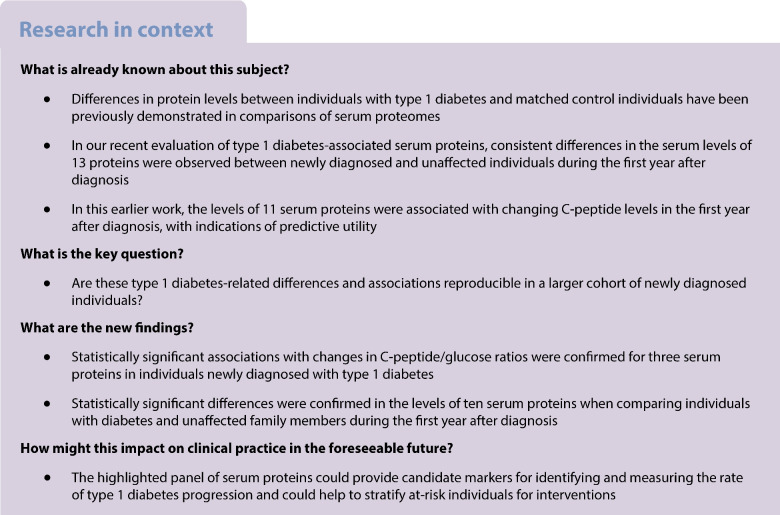


## Introduction

With growing concerns about the increasing global incidence of type 1 diabetes, there is a need for biochemical markers that can help monitor progression, treatment and remission [1]. As part of the INNODIA study (Innovative approaches to understanding and arresting type 1 diabetes), we previously demonstrated serum protein differences between newly diagnosed (ND) individuals in the first year after diagnosis and unaffected family members (UFMs), together with statistically significant associations between serum proteins and C-peptide levels [2]. As a follow-up to the initial investigation, we analysed these proteins in samples from a separate and larger group of ND youth (*n*=146; 560 samples) and UFMs (*n*=272) who were subsequently recruited for the study.

## Methods

### Samples

Sera were collected from autoantibody-positive ND individuals with type 1 diabetes who were consecutively recruited to the INNODIA study. In keeping with our previous study [2], samples from individuals diagnosed under the age of 18 years were selected for analysis (*n*=146; 91 male and 55 female participants). Sex was based on reporting by parents or adult study participants. No selection restrictions were applied concerning regional or socioeconomic factors. Accurate data on ethnicity were not available and no specific ethnicity criteria were applied. Additional samples were collected from autoantibody-negative UFMs (*n*= 272; 169 male and 103 female participants) with a similar age range (see electronic supplementary material [ESM] Table 1, ESM Fig. 1). The ND samples were collected within the first 6 weeks of diagnosis (*n*=146) and then 3 months (*n*=132), 6 months (*n*=138) and 12 months (*n*=144) after diagnosis. Only one sample was collected from each UFM.

The study followed the guidelines of the Declaration of Helsinki for research on human participants, and the study protocols were approved by the ethics committees of the participating hospitals. Either the parents or participants gave their written informed consent.

### Fasting C-peptide and fasting glucose measurements

As a surrogate measurement for beta cell function, fasting C-peptide and fasting serum glucose were measured as previously described [3, 4]. Decreasing C-peptide/glucose ratios were interpreted as an indication of likely disease progression [4].

### Sample preparation and targeted LC-MS/MS

Serum samples were prepared and analysed as previously described with slight modifications, as detailed in ESM Methods. In brief, sera were digested with trypsin, spiked with isotope-labelled analogues of the targeted peptides and analysed by selected reaction monitoring (SRM) using a TSQ Vantage Triple Quadrupole Mass Spectrometer (Thermo Scientific, USA), coupled with an Evosep One liquid chromatograph (Evosep, Denmark).

### Data analysis

#### Pre-processing, normalisation and false discovery rate calculations

Data analysis was conducted as previously described [2]. Briefly, linear mixed models (LMMs) were used to normalise the log_2_-transformed peptide abundances, adjusting for acquisition batch and run order. Periodic analysis of quality control samples indicated similar data quality metrics to those in our earlier study.

LMMs were performed using R version 4.0.0 [5], with the R packages lme4 version 1.1–27.1 and lmerTest version 3.1-3 [6].

#### Changing peptide levels and beta cell function

Regression analysis on the natural logarithm-transformed fasting C-peptide/glucose ratios during the first year from diagnosis was carried out using LMMs adjusted for sex, height, BMI score (age-based BMI expressed as SD score), study centre and individual variation. Sex, height and BMI score were included as fixed effects, while individual and study centre were included as random effects, with individual nested under the study centre. To combine the peptide data protein-wise, meta *p* values were calculated using the sum of *z* (Stouffer’s) method with the R package metap 1.10 and adjusted for multiple correction using Benjamini–Hochberg procedure.

#### Differences in the levels of tryptic peptides measured from sera

To determine whether there were significant differences in the levels of tryptic peptides between individuals with type 1 diabetes and UFMs, peptide-wise LMMs were used. Age at baseline and sex were included as fixed effects in the LMMs, and individual and study centre were included as random effects, with individual nested under the study centre. Meta *p* values for the proteins were calculated as above.

## Results

### Apolipoprotein B-100, apolipoprotein M and glutathione peroxidase 3 are inversely associated with fasting C-peptide/glucose ratios in newly diagnosed individuals

To strengthen the verification of the previously observed associations between the target proteins and fasting C-peptide/glucose ratios [2], additional peptides were included in the analysis. Significant inverse associations (*p*<0.05) with fasting C-peptide/glucose ratios were demonstrated for two peptides from each of glutathione peroxidase 3 (GPX3) and apolipoprotein M (ApoM) and three peptides from apolipoprotein B-100 (ApoB; Table 1, ESM Table 2, ESM Fig. 2). The combined peptide data for these proteins confirmed that the effects were significant after false discovery rate (FDR) correction.

**Table 1:** Significant associations between SRM data and fasting C-peptide/glucose ratios among the proteins targeted (*n*=146 ND individuals; FDR <0.05)

Gene	Peptide sequence	Peptide effect size	Peptide *p* value	Peptide FDR	Protein meta *p* value	Protein FDR
*APOB*	EVGTVLSQVYSK	−0.13	0.025	0.096	1.2 × 10^−6^	0.025
	NIQEYLSILTDPDGK^a^	−0.23	4.0 × 10^−4^	0.005		
	ITENDIQIALDDAK^a^	−0.26	0.002	0.019		
*APOM*	AFLLTPR	−0.45	3.2 × 10^−4^	0.005	1.3 × 10^−5^	7.0 × 10^−5^
	SLTSCLDSK^a^	−0.25	0.006	0.038		
*GPX3*	FLVGPDGIPIMR	−0.20	0.025	0.096	0.0018	0.0068
	QEPGENSEILPTLK^a^	−0.27	0.024	0.096		
	NSCPPTSELLGTSDR^a^	–0.16	0.14	0.45		

Effect sizes and FDRs for the selected peptides are shown, along with the combined protein meta *p* values and FDRs. The full results, together with the values from our previous study [2], are shown in ESM Table 2

^a^Peptides not measured in our previous study [2]

### Comparison of ND individuals and UFMs demonstrates differences in peptide levels during the first year after diagnosis

The comparisons of peptide levels between ND individuals and UFMs verified most of our earlier findings [2]. The results included comparable significant differences in the levels of 19 peptides, representing ten proteins (*p*<0.05; Table 2, ESM Table 3, ESM Figs 3, 4). Furthermore, the significant peptides included four of the additional peptides that were included in the analysis for hepatocyte growth factor activator (HGFAC), haemoglobin subunit beta (HBB) and GPX3. The combined peptide data further demonstrated that the differences were significant for these proteins after FDR correction.

**Table 2:** Significant differences in levels of targeted proteins between ND individuals (*n*=146) and UFMs (*n*=272) (FDR<0.05)

Gene	Peptide sequence	Peptide effect size	Peptide *p* value	Peptide FDR	Protein meta *p* value	Protein FDR
*AFM*	DADPDTFFAK	−0.14	5.8 × 10^−7^	3.1 × 10^−6^	1.9 × 10^−7^	3.7 × 10^−7^
	GQCIINSNK	−0.19	0.016	0.025		
	AESPEVCFNEESPK	−0.08	0.037	0.049		
*APOC1*	EWFSETFQK	−0.26	4.7 × 10^−4^	0.001	6.4 × 10^−6^	9.6 × 10^−6^
	EFGNTLEDK	−0.16	0.002	0.004		
*C2*	HAFILQDTK	0.14	1.1 × 10^−5^	4.1 × 10^−5^	8.3 × 10^−10^	2.5 × 10^−9^
	AVISPGFDVFAK	0.14	9. 6 × 10^−6^	4.1 × 10^−5^		
*F2*	TATSEYQTFFNPR	0.42	6.4 × 10^−26^	1.7 × 10^−24^	3.2 × 10^−17^	1.9 × 10^−16^
*GPX3*	NSCPPTSELLGTSDR^a^	0.14	4.0 × 10^−5^	1.4 × 10^−4^	2.5 × 10^−9^	6.1 × 10^−9^
	QEPGENSEILPTLK^a^	0.09	2.9 × 10^−4^	8.6 × 10^−4^		
	FLVGPDGIPIMR	0.09	0.003	0.006		
*HBB*	VNVDEVGGEALGR^a^	0.36	5.2 × 10^−4^	0.001	2.6 × 10^−6^	4.4 × 10^−6^
	SAVTALWGK	0.35	7.7 × 10^−4^	0.002		
*HGFAC*	LEACESLTR	0.3	7.6 × 10^−11^	6.9 × 10^−10^	2.3 × 10^−10^	9.0 × 10^−10^
	VANYVDWINDR^a^	0.14	0.008	0.014		
*HRG*	DGYLFQLLR	0.1	0.012	0.021	0.0038	0.0051
	ADLFYDVEALDLESPK^a^	0.08	0.064	0.083		
*TGFBI*	LTLLAPLNSVFK	−0.08	0.016	0.025	0.016	0.018
*TTR*	AADDTWEPFASGK	−0.28	1.4 × 10^−11^	1.9 × 10^−10^	3.1 × 10^−18^	3.7 × 10^−17^
	TSESGELHGLTTEEEFVEGIYK	−0.3	1.4 × 10^−8^	9.6 × 10^−8^		

Effect sizes and FDRs for the selected peptides are shown, along with the combined protein meta *p* values and FDRs. The full results, together with the values from our previous study [2], are shown in ESM Table 3

^a^Peptides not measured in our previous study [2]

## Discussion

Following on from our earlier study of serum protein markers of type 1 diabetes in youth among the first 100 ND participants in the INNODIA study [2], we have now analysed sera from the next 150 ND individuals recruited to the study. The previously reported inverse associations with fasting C-peptide/glucose ratios were confirmed for targeted peptides from the proteins ApoB, ApoM and GPX3. Furthermore, we verified the majority of the peptide-level differences between ND individuals and UFMs reported previously [2].

One of the challenges in serum proteomics is the pre-analytical variability, which can impact protein quantification [7, 8]. However, despite these challenges, the validations of the protein differences between ND individuals and UFMs were highly consistent. In this respect, our study benefited from a tightly controlled longitudinal sample collection protocol, along with the inclusion of matched UFMs. With recruitment based on diagnosis of type 1 diabetes, there were, however, more male than female participants in the cohort, following the tendency for a higher frequency of type 1 diabetes in male than female populations [9]. Nevertheless, for both the comparisons of peptide measurements between ND individuals and UFMs and the analysis of associations between fasting C-peptide/glucose ratios and target proteins, sex was included in the LMMs. This inclusion supports the generalisability of the findings to both male and female populations.

Although the results from the ND and UFM comparison were mostly validated, the peptide and C-peptide/glucose associations were consistent for only three of the 11 previously reported target proteins [2]. Notably, the parabolic course of the fasting C-peptide/glucose ratios in the first year was less pronounced in the follow-up data than in our previous study [2] (ESM Fig. 5).

Insulin plays a vital role in the regulation of lipid metabolism and lipid disorders are commonly diagnosed in individuals with type 1 diabetes [10]. In this follow-up study, we confirmed the inverse associations between fasting C-peptide/glucose ratios and peptides from ApoB and ApoM. Both of these proteins are expressed extensively in the major sites of insulin action, including liver and adipose tissue [10, 11]. Insulin indirectly inhibits ApoB-containing triacylglycerol-rich lipoprotein production and promotes clearance of the particles [10]. Interestingly, although ApoM is primarily produced in hepatocytes, it is also formed in human adipose tissue, and adipose ApoM levels have been reported to correlate positively with plasma ApoM levels [11]. Certain SNPs of ApoM have been associated with type 1 diabetes [12]. Additionally, ApoM has been shown to increase insulin secretion by binding to bioactive sphingolipid sphingosine 1-phospate (S1P) [13] and, in turn, insulin has been shown to inhibit ApoM expression [14]. Therefore, the inverse relationship between fasting C-peptide/glucose ratios and peptides from ApoB and ApoM may reflect how changes in endogenous insulin affect the secretion of both proteins and the clearance of ApoB-containing particles. Additionally, our data confirm the lower levels of apolipoprotein C1 (ApoC1) in sera from ND individuals compared with UFMs. Interestingly, recent reports have demonstrated that plasma/serum ApoC1 levels are already reduced after seroconversion [7, 15].

In keeping with our earlier study [2], and putatively indicative of oxidative stress, three peptides from GPX3 were detected at higher levels in ND individuals than in UFMs (Table 2). The previously noted inverse association with fasting C-peptide/glucose ratios observed for GPX3 [2] was significant for two of the measured peptides (*p*<0.05; Table 1). As before, and noted in relation to its role in the transport of the antioxidant vitamin E, the three peptides measured for afamin (AFM) were less abundant in ND individuals than in UFMs.

Consistent with our previous results [2], peptides from TGF-beta-induced protein ig-h3 (TGFBI) and transthyretin (TTR) were less abundant in ND individuals. These two proteins have been associated with islet cell survival and beta cell integrity, respectively [16], and their lower levels could signify how this milieu is compromised in type 1 diabetes.

Lastly and consistent with our previous study [2], coagulation factors and complement-related proteins were found at higher levels in ND individuals (Table 2). The increased levels of prothrombin (F2) and HGFAC may reflect thrombotic differences manifested in ND individuals [17].

### Summary

These analyses confirm the previously reported differences in relative levels of proteins associated with type 1 diabetes between individuals with type 1 diabetes and unaffected individuals. These data, together with relationships between the target proteins and C-peptide/glucose ratios, further highlight the importance of these proteins, together with their associated pathways, in the pathogenesis of type 1 diabetes. Further research is needed to explore the clinical value of these targets and how these findings reflect and contribute to underlying disease pathogenesis.

## Electronic supplementary material

Below is the link to the electronic supplementary material.

Supplementary file1 (PDF 1802 KB)

## Data Availability

Access to these person-sensitive data is only through secure environment by application to the INNODIA Data Access Committee (see https://www.innodia.eu/).

## Appendix

**Members of the INNODIA and INNODIA HARVEST consortia**

C. Mathieu, P. Gillard, K. Casteels, L. Overbergh (KU Leuven, Belgium); D. Dunger, C. Wallace, M. Evans, A. Thankamony, E. Hendriks, S. Bruggraber, A. Qureshi, L. Marcovecchio, Paediatrics laboratory staff (University of Cambridge, UK); M. Peakman, T. Tree (King’s College London, UK); N. Morgan, S. Richardson (University of Exeter, UK), J. Todd, L. Wicker (University of Oxford, UK); A. Mander, C. Dayan, M. Alhadj Ali (Cardiff University, UK); T. Pieber (Medical University of Graz, Austria); D. Eizirik, M. Cnop (Université Libre de Bruxelles, Belgium); S. Brunak (University of Copenhagen, Denmark); F. Pociot, J. Johannesen, P. Rossing, C. Legido Quigley (Herlev University Hospital, Region Hovedstaden, Denmark); R. Mallone, R. Scharfmann, C. Boitard (Cochin Institute Paris, France); M. Knip, T. Otonkoski, T. Vatanen (University of Helsinki, Finland); R. Veijola (University of Oulu, Finland); R. Lahesmaa, M. Oresic, J. Toppari (University of Turku, Finland); T. Danne (Children’s and Youth Hospital Hannover, Germany); A. G. Ziegler, P. Achenbach, T. Rodriguez-Calvo (Helmholtz Zentrum Muenchen, Germany); M. Solimena, E. Bonifacio, S. Speier (TU Dresden, Germany); R. Holl (University of Ulm, Germany); F. Dotta (University of Siena, Italy); F. Chiarelli (University of Chieti, Italy); P. Marchetti (University of Pisa, Italy); E. Bosi (University Vita-Salute San Raffaele, Italy); S. Cianfarani, P. Ciampalini (Bambino Gesù Children’s Hospital, Italy); C. de Beaufort (Centre Hospitalier de Luxembourg, Luxemburg); K. Dahl-Jørgensen, T. Skrivarhaug, G. Joner, L. Krogvold (Oslo University Hospital, Norway); P. Jarosz-Chobot (Medical University of Silesia, Poland); T. Battelino (University of Ljubljana, Slovenia); B. Thorens (University of Lausanne, Switzerland); M. Gotthardt (Radboud University Medical Center, the Netherlands); B. Roep, T. Nikolic, A. Zaldumbide (Leiden University Medical Center, the Netherlands); A. Lernmark, M. Lundgren (Lund University, Sweden); G. Costecalde (Univercell-Biosolutions, France); T. Strube, A. Schulte, A. Nitsche (Sanofi, Germany); M. Peakman, J. Vela (Sanofi, USA), M. von Herrath, J. Wesley (Novo Nordisk, Denmark); A. Napolitano-Rosen (GlaxoSmithKline, UK), M. Thomas, N. Schloot (Eli Lilly, UK), A. Goldfine, F. Waldron-Lynch, J. Kompa, A. Vedala, N. Hartmann, G. Nicolas (Novartis Pharma AG, Switzerland); J. van Rampelbergh, N. Bovy (Imcyse SA, Belgium); S. Dutta, J. Soderberg, S. Ahmed, F. Martin, E. Latres (Breakthrough T1D [formerly known as JDRF], USA); G. Agiostratidou, A. Koralova (The Leona M. and Harry B. Helmsley Charitable Trust, USA).

**Associated clinical sites**

R. Willemsen (Barts Health NHS Trust, UK); A. Smith (Northampton General Hospital NHS Trust, UK); B. Anand (West Suffolk NHS FT, UK); V. Puthi (North West Anglia NHS FT, UK); S. Zac-Varghese (East and North Hertfordshire NHS Trust, UK); V. Datta (Norfolk and Norwich University Hospitals NHS FT, UK); R. Dias (Birmingham Women’s and Children’s NHS FT, UK); P. Sundaram (University Hospitals of Leicester NHS Trust, UK); B. Vaidya (Royal Devon and Exeter NHS FT, UK); C. Patterson (NHS Fife, UK); K. Owen (Oxford University Hospitals NHS FT, UK); C. Dayan (Cardiff and Vale University Health Board, UK); B. Piel (Queen Elizabeth Hospital, King’s Lynn NHS FT, UK); S. Heller S (Sheffield Teaching Hospitals NHS FT, UK); T. Randell, T. Gazis (Nottingham University Hospitals NHS Trust, UK); E. Bismuth Reismen, J.-C. Carel (Hospital Robert Debre, France); J.-P. Riveline, J.-F. Gautier (Hospital Lariboisière, France); F. Andreelli (Hospital Lapitie-Salpetriere, France); F. Travert (Hospital Bichat Claude Bernard, France); E. Cosson (Hospital Jean-Verdier and Hospital Avicenne, France); A. Penfornis, C. Petit (Centre Hospitalier Sud-Francilien, France); B. Feve (Hospital St Antoine, France); N. Lucidarme (Hospital Jean-Verdier Pediatrie, France);); J.-P. Beressi (Hospital André Mignot, France); C. Ajzenman (Hospital André Mignot Pediatrie, France); A. Radu (Hospital Européen Georges-Pompidou, France); S. Greteau-Hamoumou (Hospital Louis Mourier, France); C. Bibal (Hospital Kremlin Bicêtre, France); T. Meissner (Universitätsklinikum der Heinrich-Heine-Universität Düsseldorf, Germany); B. Heidtmann (Katholisches Kinderkrankenhaus Wilhelmstift, Germany); S. Toni (AOU Meyer, Italy); B. Rami-Merhar (Medical University of Vienna, Austria); B. Eeckhout, B. Peene, N. Vantongerloo (Algemeen Ziekenhuis Geel Sint-Dimpna Geel, Belgium); T. Maes, L. Gommers (Imeldziekenhuis Bonheiden, Belgium).

## Abbreviations

ApoB: Apolipoprotein B-100
ApoC1: Apolipoprotein C1
ApoM: Apolipoprotein M
FDR: False discovery rate
GPX3: Glutathione peroxidase 3
HGFAC: Hepatocyte growth factor activator
INNODIA: Innovative approaches to understanding and arresting type 1 diabetes
LMM: Linear mixed model
ND: Newly diagnosed
SRM: Selected reaction monitoring
UFM: Unaffected family member
