# Supplementary material for: Targeted serum proteomics of longitudinal samples from newly diagnosed youth with type 1 diabetes affirms markers of disease
Source: Diabetologia. 2025 Feb 28;68(6):1108–14. doi: 10.1007/s00125-025-06394-7 (PMC12069125; doi:10.1007/s00125-025-06394-7)
Supplement: Supplementary file 1 — Supplementary file1 (PDF 1802 KB) [file 125_2025_6394_MOESM1_ESM.pdf]

SUPPLEMENTARY INFORMATION

Targeted serum proteomics of longitudinal samples from newly diagnosed youth with type 1 diabetes affirms markers of disease

Robert Moulder (0000-0003-4742-0566) [1,2 †], M. Karoliina Hirvonen, (0000-0003-1575-9725)[1,2*], Tommi Välikangas (0000-0002-6046-1920)[1,2*], Tomi Suomi (0000-0003-3639-979X)[1,2], Lut Overbergh (0000-0001-7126-356X)[3], Mark Peakman (0000-0003-3328-3513)[4], Soren Brunak (0000-0003-0316-5866)[5], Chantal Mathieu (0000-0002-4055-5233)[3], Mikael Knip, (0000-0003-0474-0033)[6,7,8], Laura L. Elo (0000-0001-5648-4532)[1,2,9 †], Riitta Lahesmaa (0000-0002-4782-9567)[1,2,9 †]

On behalf of the INNODIA Consortium [#]

[1] Turku Bioscience Centre, University of Turku and Åbo Akademi University, Turku, Finland

[2] InFLAMES Research Flagship Centre, University of Turku, Turku Finland

[3] Katholieke Universiteit Leuven/ Universitaire Ziekenhuizen, Leuven, Belgium.

[4] Immunology & Inflammation Research Therapeutic Area, Sanofi, Cambridge, UK,

[5] Novo Nordisk Foundation Centre for Protein Research, Faculty of Health and Medical Sciences, University of Copenhagen, Copenhagen, Denmark

[6] Research Program for Clinical and Molecular Metabolism, Faculty of Medicine, University of Helsinki, Helsinki, Finland

[7] Pediatric Research Centre, New Children's Hospital, Helsinki University Hospital, Helsinki, Finland

[8] Department of Pediatrics, Tampere University Hospital, Tampere, Finland

[9] Institute of Biomedicine, University of Turku, Turku, Finland

[†] Corresponding authors:

Robert Moulder: robmou@utu.fi

Riitta Lahesmaa: rilahes@utu.fi,

Laura L Elo: laura.elo@utu.fi

INNODIA - "Innovative approaches to understanding and arresting type 1 diabetes": https://www.innodia.eu/

Contents

ESM Methods 3
Sample preparation 3
Targeted LC-MS/MS 3
ESM Table 1: Samples compared in this study and the site of their collection 4
ESM Table 2: The comparison of targeted proteomics and fasting C-peptide/glucose associations in the “first 100” and the subsequent “next 150” INNODIA (current study)... 5
ESM Table 3: Targeted proteomics results comparing the ND and AAb- UFMs from both the “first 100” and subsequent “next 150” INNODIA patients 6
ESM Fig. 1: The age and sex distribution of the samples used in this study. 7
ESM Fig. 2: Peptide associations with the fasting C-peptide/glucose ratio were reiterated for three proteins. 8
ESM Fig. 3. Comparison of the targeted proteomics data from the age-matched ND and AAb- UFMs confirmed several peptides with increased serum levels in ND. 9
ESM Fig. 4. Comparison of the targeted proteomics data from the age-matched ND and AAb- UFMs confirmed several peptides with decreased serum levels in ND. 10
ESM Fig. 5. Comparison of the fasting C-peptide/glucose characteristic of the ND individuals from the “first 100” and “next 150” from the INNODIA study: 11
References 11

ESM Methods

Sample preparation

The serum samples were prepared in eleven batches, divided among eleven 96-well plates. Three distinct quality control (QC) serum samples were included in the preparation of each plate. The plates were designed to include newly diagnosed (ND) individuals, along with sex- and age-matched unaffected family members (UFMs), with the order among the matched sample groups randomised across the plates.

Serum (4 µl) was diluted with 8M urea in Tris-HCl (100 µl x 50 mM). The samples were then processed using a Biomek^{NX} robot as follows: The samples were reduced in 10 mM DTT (1-hour, 37 ˚C), and alkylated with 10 mM IAA in darkness (30 min, room temperature). Half the sample (55 µl) was aliquoted and diluted with Tris-HCl (400 µl x 50 mM), digested with trypsin (4.3 µg) with an estimated 1:30 ratio of trypsin: protein at 37 ^{0}C overnight, then quenched with 10% TFA. The digests were desalted using 96 well SepPak 100 mg reversed phase SPE plates (SepPak C18, Waters, SKU: 186002321), as previously described [1, 2]

The desalted sample were dried using a vacuum centrifuge (SAVANT SPD1010, Thermo Scientific), reconstituted in MilliQ water containing 2% formic acid, 0.1% acetonitrile, and the peptide recovery estimated by absorbance at 280 nm using a NanoDrop-1000 UV spectrophotometer (Thermo Scientific). The samples were diluted to 80 ng/µl, then 1:1 spiked solution of heavy isotope-labelled synthetic peptides analogues spiked (~10fmol/µl, typically two peptides per protein, PEPotec, Thermo Fischer Scientific) and retention time standards (MSRT1, Sigma).

Targeted LC-MS/MS

Skyline software [3] was used to develop the retention time-scheduled data acquisition method and subsequently check the peak detection and integration

Retention time scheduled selected reaction monitoring (SRM) mass spectrometry (MS) was used as previously described [1]. In brief, a TSQ Vantage Triple Quadrupole Mass Spectrometer (Thermo Scientific) was coupled with an Evosep One liquid chromatograph (Evosep, Denmark). The 60 sample per day (24 minute) method was used with an EV1094 Endurance 80 mm x 150 µm i.d. analytical column packed with 3 µm C18-bonded silica (Dr Maisch GmbH).

The estimated peptide amount analysed was 800 ng, based on 20 µl x 0.04 µg/µl loaded onto Evotip Pure disposable trap columns (Evosep, Denmark), according to the manufacturer’s instructions.

Both the heavy and light peptides were measured throughout. The elution profiles were compared with the heavy peptides and the peak boundaries corrected where appropriate. Transitions that could not be consistently corrected to ensure profiles matching the synthetic peptide were removed.

SUPPLEMENTARY INFORMATION

ESM Table 1: Samples compared in this study and the site of their collection.

Center	ND (n)	ND age range (medium)	AAb- UFM (n)	UFM age range (medium)
Ziekenhus Geel, Belgium	0	NA	4	10 (1-12)
CHL - Centre Hospitalier de Luxembourg	19	8 (2-16)	15	8 (2-16)
Herlev University Hospital, Denmark	12	12 (8-17)	16	12 (8-17)
UH- Meyer, Florenz, Italy	0	NA	1	7 (7-7)
Ospedale Pediatrico Bambino Gesu, Rome, Italy	5	10 (7-14)	2	11 (10-12)
KU Leuven, Belgium	7	11 (1-14)	31	11 (1-17)
Lund University, Sweden	0	NA	7	12 (9-16)
Medical University of Graz, Austria	6	13.5 (3-17)	20	10.5 (3-16)
Medical University Vienna, Austria	0	NA	8	9 (4-15)
Oslo Universitetssykehus HF, Norway	0	NA	1	14 (14-14)
Slaski Uniwersytet Medyczny w Katowicach, Poland	9	9 (5-15)	18	9 (5-15)
University of Cambridge, UK	6	13.5 (9-17)	9	13 (2-17)
University of Helsinki, Finland	42	8 (1-15)	67	8 (1-15)
Barts Health NHS Trust, London, UK	2	2 (2-2)	0	NA
East & North Herts Trust - Lister, UK	0	NA	3	9 (9-9)
Leicester Royal Infirmary, UK	2	9.5 (9-10)	0	NA
North West Anglia (Peterborough), UK	0	NA	1	2 (2-2)
Northampton General Hospital, UK	0	NA	5	14 (10-14)
Birmingham Children’s Hospital, UK	0	NA	2	7 (2-12)
Universite Libre de Bruxelles, Belgium	2	15 (13-17)	5	13 (7-17)
University of Ljubljana, Slovenia	20	10 (5-15)	18	9.5 (5-17)
Universita degli Studi di Siena, Italy	0	NA	3	7 (6-8)
San Raffaele Hospital, Italy	0	NA	5	14 (9-15)
Oulun University, Finland	14	8.5 (2-16)	25	9 (2-16)
Turku University, Finland	0	NA	6	8 (1-15)
Male (total)	91		169	
Female (total)	55		103	

ESM Table 2: The comparison of targeted proteomics and fasting C-peptide/glucose associations in the “first 100” and the subsequent “next 150” INNODIA (current study). The results from the two groups were not combined for the statistical analysis. The FDRs and effects size are displayed. Additional peptides measured in the follow-up analyses are marked NA in the “first 100”, and the FDR significant sequnces highlighted in bold text. In addition, a synthetic oxidized methionine peptide was included and monitored for FLVGPDGIPIM[Oxi]R (from GPX3) to rule out any bias created by oxidation of this residue.

**Gene | Accession**	**Peptide**	**First 100 Effect Size**	**First 100 p-value**	**First 100 FDR**	**Next 150 Effect Size**	**Next 150 p-value**	**Next 150 FDR**
APOB | P04114	EVGTVLSQVYSK	-0.22	0.002	0.002	-0.129	0.025	0.096
APOB | P04114	**NIQEYLSILTDPDGK**	NA	NA	NA	-0.229	0.0004	0.005
APOB | P04114	**ITENDIQIALDDAK**	NA	NA	NA	-0.259	0.0021	0.019
APOB | P04114	ENFAGEATLQR	NA	NA	NA	0.059	0.22	0.499
APOB | P04114	EYSGTIASEANTYLNSK	NA	NA	NA	0.0099	0.79	0.902
APOL1 | O14791	VTEPISAESGEQVER	0.51	0.003	0.003	0.043	0.61	0.821
APOM | O95445	AFLLTPR	-0.52	0.003	0.003	-0.448	0.00032	0.005
APOM | O95445	**SLTSCLDSK**	NA	NA	NA	-0.25	0.0056	0.038
C8G | P07360	SLPVSDSVLSGFEQR	-0.53	0.002	0.002	-0.01	0.21	0.499
C8G | P07360	VQEAHLTEDQIFYFPK	NA	NA	NA	-0.089	0.37	0.643
C8G | P07360	SLPVSDSVLSGFEQR	NA	NA	NA	0.02	0.83	0.902
COL1A1 | P02452	ICVCDNGK	0.22	0.002	0.002	-0.018	0.44	0.694
COL1A1 | P02452	VLCDDVICDETK	NA	NA	NA	0.029	0.54	0.774
GPX3 | P22352	FLVGPDGIPIMR	-1.04	0	0	-0.204	0.025	0.096
GPX3 | P22352	QEPGENSEILPTLK	NA	NA	NA	-0.267	0.024	0.096
GPX3 | P22352	NSCPPTSELLGTSDR	NA	NA	NA	-0.16	0.14	0.45
GPX3 | P22352	FLVGPDGIPIM[Oxi]R	NA	NA	NA	-0.077	0.369	0.643
HRG | P04196	DGYLFQLLR	-0.42	0.003	0.003	-0.038	0.716	0.879
HRG | P04196	ADLFYDVEALDLESPK	NA	NA	NA	0.001	0.989	0.989
IGF1 | P05019	GFYFNKPTGYGSSSR	0.31	0	0	-0.006	0.827	0.902
IGF1 | P05019	APQTGIVDECCFR	NA	NA	NA	0.015	0.381	0.643
IGFBP2 | P18065	LEGEACGVYTPR	-0.27	0.001	0.001	-0.034	0.218	0.499
IGFBP2 | P18065	LIQGAPTIR	-0.28	0.001	0.001	-0.047	0.356	0.643
MASP2 | O00187	VLATLCGQESTDTER	-0.36	0.001	0.001	-0.099	0.15	0.45
MASP2 | O00187	LASPGFPGEYANDQER	NA	NA	NA	0.007	0.906	0.941
SERPING1 | P05155	LLDSLPSDTR	-0.64	0.001	0.001	-0.076	0.537	0.774
SERPING1 | P05155	TLYSSSPR	NA	NA	NA	0.027	0.695	0.879

ESM Table 3: Targeted proteomics results comparing the ND and AAb- UFMs from both the “first 100” and subsequent “next 150” INNODIA patients (n =86 and 146 ND and n= 172 and 272 UFM). The FDRs and effects size are displayed. Additional peptides measured in the follow-up analyses are marked NA in the “first 100”, and the FDR significant sequnces highlighted in bold text. The results from the two groups were not combined for the statistical analysis.

**Gene | Accession**	**Peptide**	**First 100 Effect Size**	**First 100 P-value**	**First 100 FDR**	**Next 150 Effect Size**	**Next 150 p-value**	**Next 150 FDR**
AFM | P43652	DADPDTFFAK	-0.13	3.4E-05	5.5E-04	-0.14	5.8E-07	3.1E-06
AFM | P43652	GQCIINSNK	-0.2	1.3E-05	2.6E-04	-0.19	0.016	0.025
AFM | P43652	AESPEVCFNEESPK	-0.16	3.2E-06	7.1E-05	-0.08	0.037	0.049
APOC1 | P02654	EWFSETFQK	-0.46	2.7E-09	1.2E-07	-0.26	4.7E-04	0.001
A*POC1* | P02654	EFGNTLEDK	-0.42	1.4E-10	8.5E-09	-0.16	0.002	0.004
C2 | P06681	HAFILQDTK	0.13	0.0037	1.2E-05	0.14	1.1E-05	4.1E-05
C2 | P06681	AVISPGFDVFAK	0.08	4.9E-07	0.039	0.14	9.6E-06	4.1E-05
F2 | P00734	TATSEYQTFFNPR	0.26	1.1E-10	8.5E-09	0.42	6.4E-26	1.7E-24
F2 | P00734	YGFYTHVFR	NA	NA	NA	-0.04	0.088	0.11
GPX3 | P22352	**NSCPPTSELLGTSDR**	NA	NA	NA	0.14	4.0E-05	1.4E-04
GPX3 | P22352	**QEPGENSEILPTLK**	NA	NA	NA	0.09	2.9E-04	8.6E-04
GPX3 | P22352	FLVGPDGIPIMR	0.11	3.7E-04	0.006	0.09	0.003	0.006
GPX3 | P22352	FLVGPDGIPIM[Oxi]R	NA	NA	NA	0.03	0.32	0.35
HBB | P68871	**VNVDEVGGEALGR**	NA	NA	NA	0.36	5.2E-04	0.001
HBB | P68871	SAVTALWGK	0.4	0.005	0.047	0.35	7.7E-04	0.002
HGFAC | Q04756	LEACESLTR	0.14	9.6E-04	0.013	0.3	7.6E-11	6.9E-10
HGFAC | Q04756	**VANYVDWINDR**	NA	NA	NA	0.14	0.008	0.014
HRG | P04196	DGYLFQLLR	0.16	0.001	0.017	0.1	0.012	0.021
HRG | P04196	ADLFYDVEALDLESPK	NA	NA	NA	0.08	0.064	0.083
IGF1 | P05019	APQTGIVDECCFR	-0.35	0.003	0.038	-0.14	0.35	0.36
IGF1 | P05019	GFYFNKPTGYGSSSR	NA	NA	NA	0.04	0.69	0.69
IGFBP3 | P17936	ETEYGPCR	-0.11	2.7E-07	0.03	0.09	0.036	0.049
IGFBP3 | P17936	SAGSVESPSVSSTHR	NA	NA	NA	0.09	0.092	0.11
IGFBP3 | P17936	FLNVLSPR	NA	NA	NA	0.04	0.13	0.15
TGFBI | Q15582	LTLLAPLNSVFK	-0.22	NA	4.0E-04	-0.08	0.016	0.025
TTR |P02766	AADDTWEPFASGK	-0.44	1.2E-10	8.5E-09	-0.28	1.4E-11	1.9E-10
TTR | P02766	TSESGELHGLTTEEEFVEGIYK	-0.46	1.2E-08	4.2E-07	-0.30	1.4E-08	9.6E-08

SUPPLEMENTARY INFORMATION

ESM Fig. 1: The age and sex distribution of the samples used in this study.

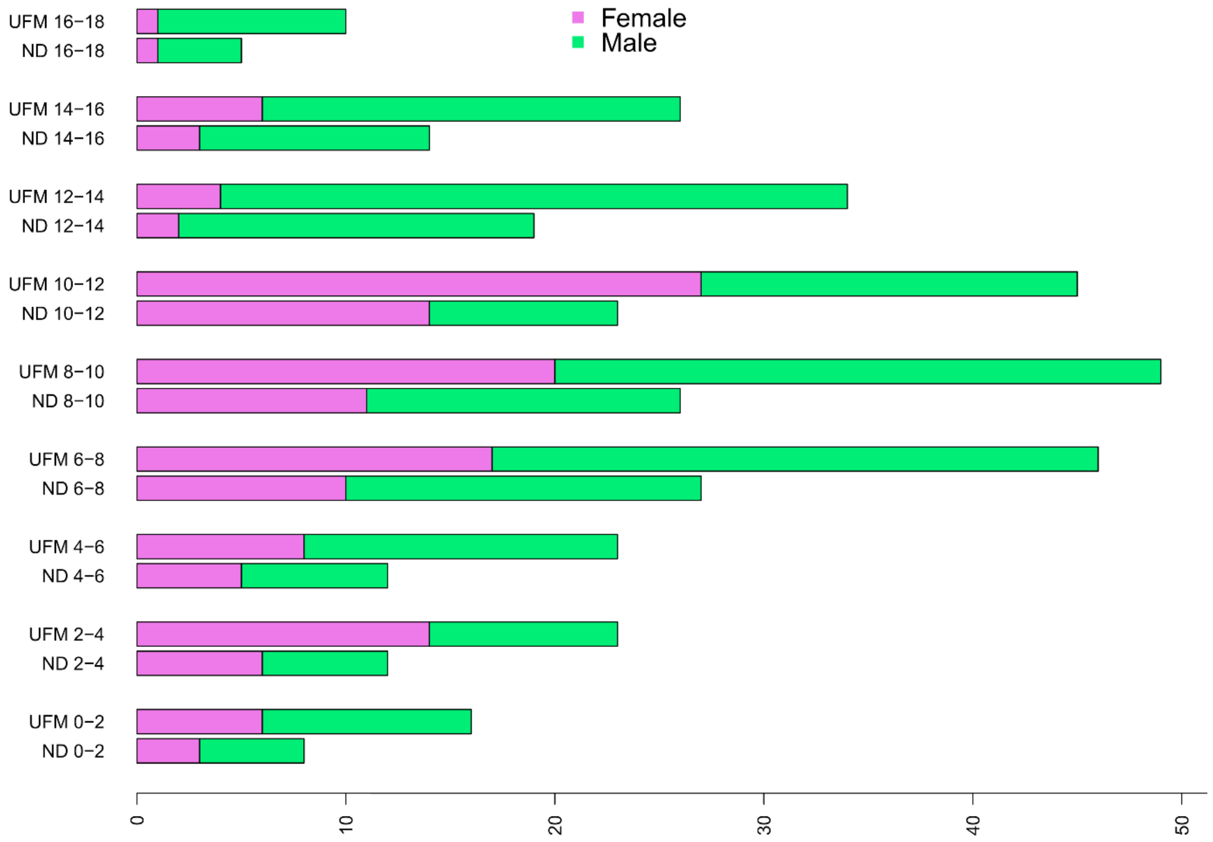

ESM Fig. 2: Peptide associations with the fasting C-peptide/glucose ratio were reiterated for three proteins. These included peptides from (a) ApoB, (b) ApoM and (c) GPX3. The data is represented as local regression (loess) smoothing curves for the normalized peptide abundances (red) and the C-peptide/glucose ratios (grey) from all the ND subjects relative to sampling time after disease onset. The figures with a darker background are the original results from the “first 100” study and the ones with the white background the peptides verified in the present “next 150” study. The results from the two groups were not combined for the statistical analysis. The loess curves are represented by the solid lines and their 95% confidence intervals by the dashed lines. The peptide expression data and the C-peptide/glucose ratio data were adjusted for the potential confounding factors gender, height, standardized BMI (BMI-SDS), study centre and individual variation. Both the C-peptide/glucose ratio and the peptide expression data are scaled (z-score standardized) within each feature and the scaled peptide expression values are further offset by -2 for visualization of the respective changes of both variables during the first year after the diagnosis of type 1 diabetes in the same plot. Individual data points are shown as grey for the C-peptide / glucose ratio and red for the peptide expression values to display the heterogeneity of the data. A small amount of random variation is added to the time value of each measurement for better visualization. Similarly, the scale of the Y-axis is limited between the 5th and 95th percentiles for the exclusion of extreme values for better visual presentation of the data.

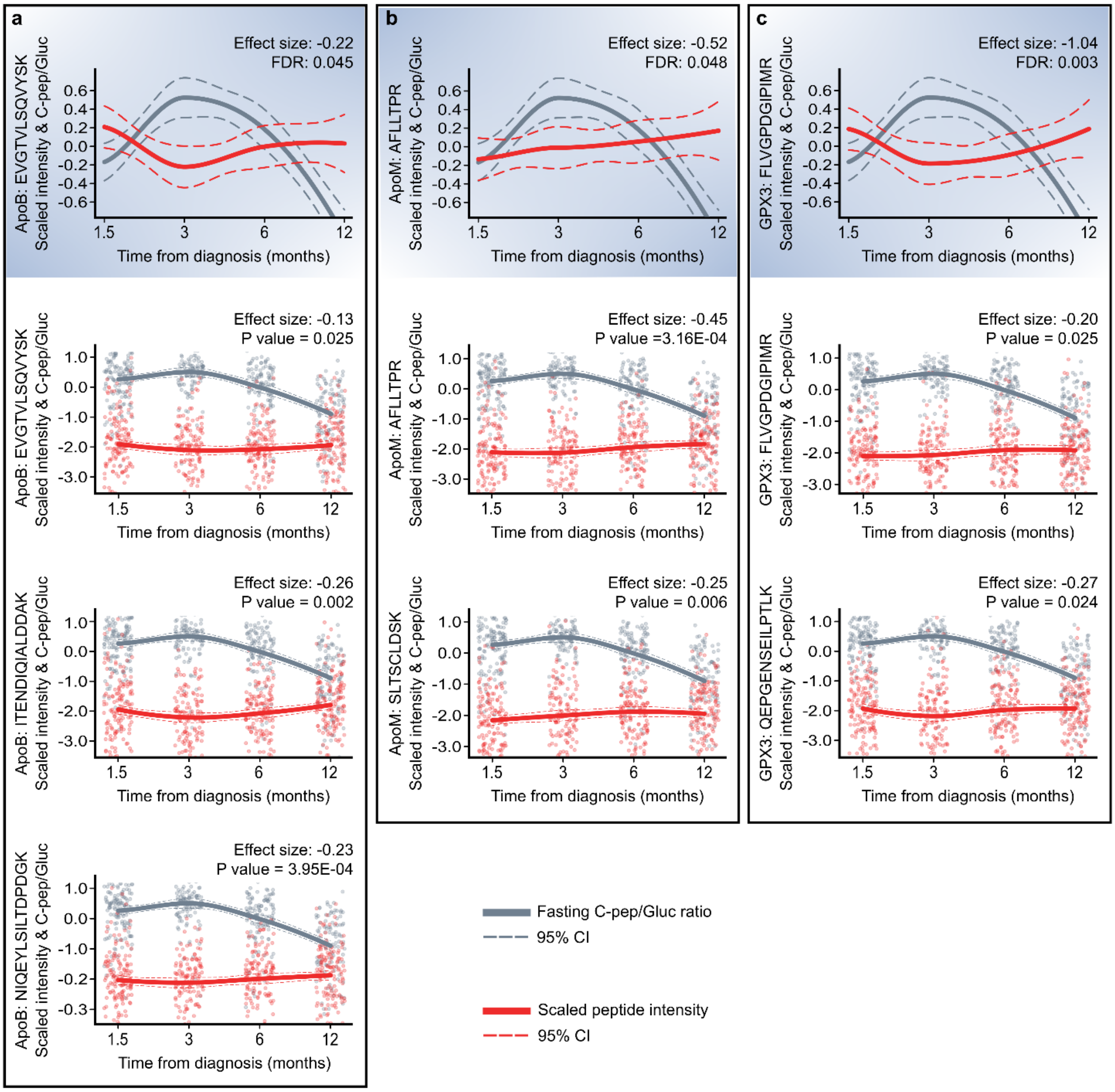

ESM Fig. 3. Comparison of the targeted proteomics data from the age-matched ND and AAb- UFMs confirmed several peptides with increased serum levels in ND. The data is represented as the local regression (loess) smoothing curves for the peptide abundances relative to age and grouped according to status, i.e. ND subjects (red) and AAb- UFMs (blue). The figures with a darker background are the original results from the “first 100” study and the ones with the white background the peptides verified in the present “next 150” study. The results from the two groups were not combined for the statistical analysis. The loess curves are represented by the solid lines and their 95% confidence intervals by the dashed lines. Individual data points are shown as red for the ND subjects and blue for the AAb- UFMs to display the heterogeneity of the data. A small amount of random variation is added to the age value of each measurement for better visualization. Similarly, the scale of the Y-axis is limited between the 5th and 95th percentiles for the exclusion of extreme values for better visual presentation of the data.

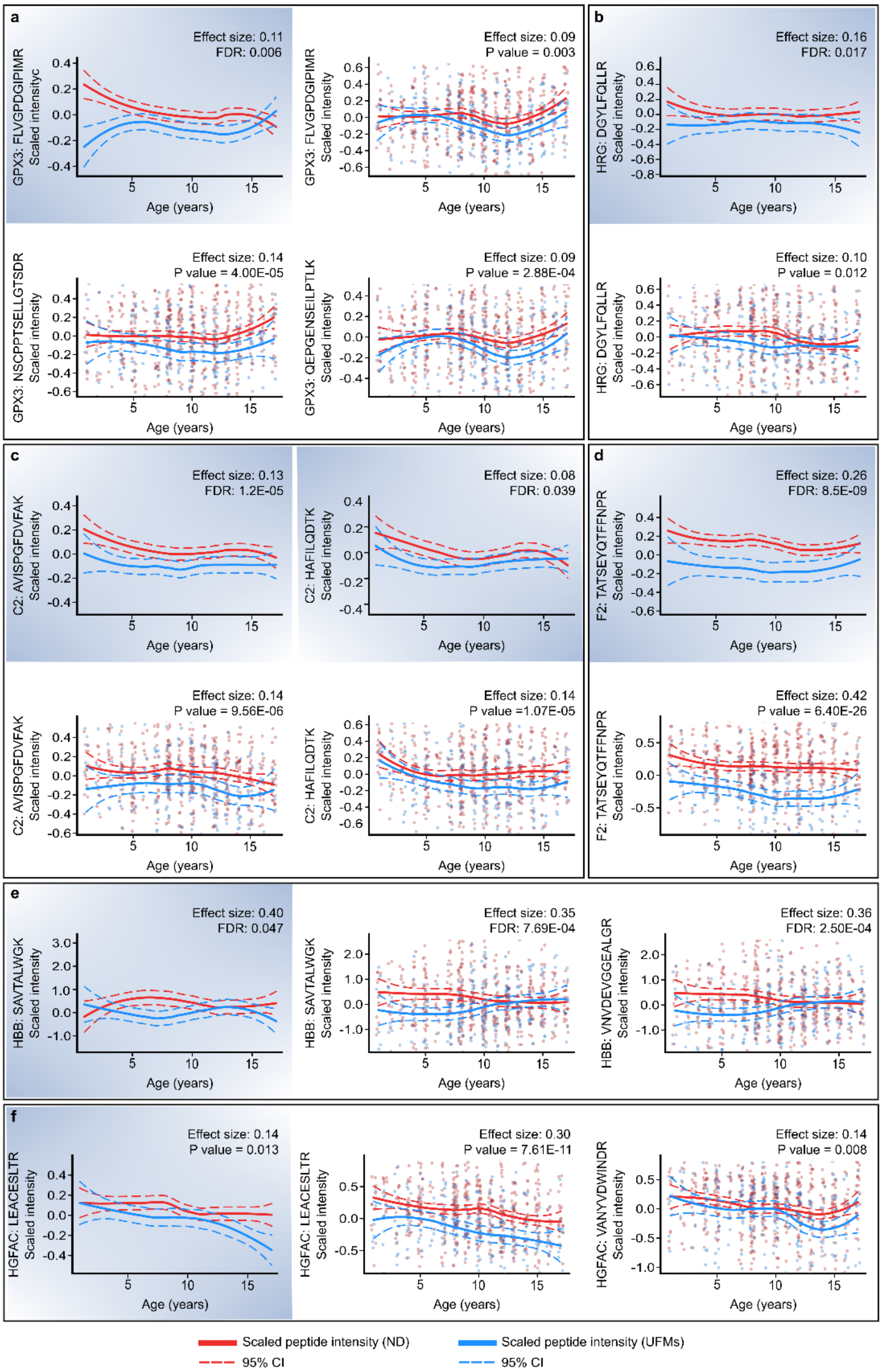

ESM Fig. 4. Comparison of the targeted proteomics data from the age-matched ND and AAb- UFMs confirmed several peptides with <u>decreased</u> serum levels in ND. The data is represented as the local regression (loess) smoothing curves for the peptide abundances relative to age and grouped according to status, i.e. ND subjects (red) and AAb- UFMs (blue). The figures with a darker background are the original results from the “first 100” study and the ones with the white background the peptides verified in the present “next 150” study. The results from the two groups were not combined for the statistical analysis. The loess curves are represented by the solid lines and their 95% confidence intervals by the dashed lines. Individual data points are shown as red for the ND subjects and blue for the AAb- UFMs to display the heterogeneity of the data. A small amount of random variation is added to the age value of each measurement for better visualization. Similarly, the scale of the Y-axis is limited between the 5th and 95th percentiles for the exclusion of extreme values for better visual presentation of the data.

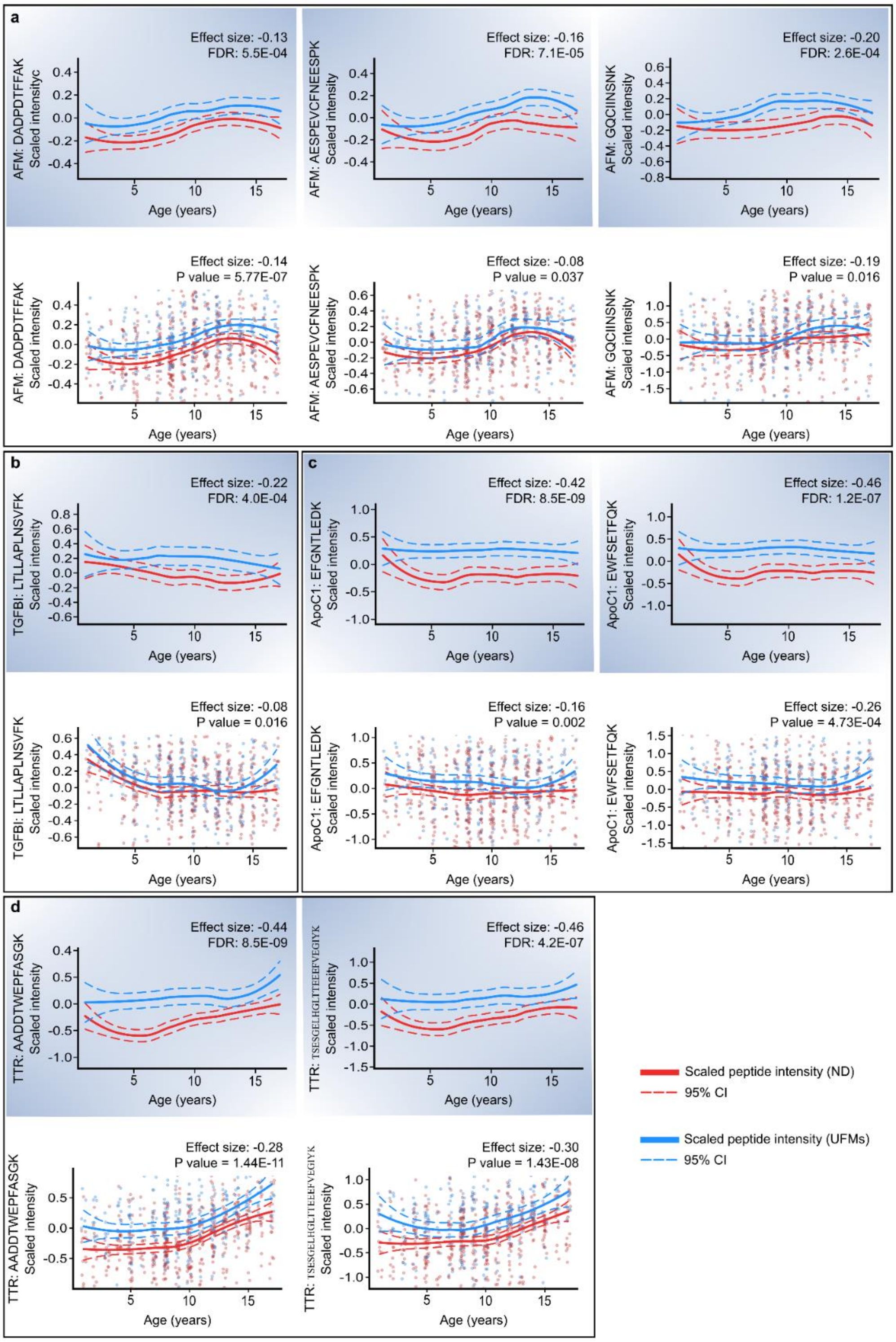

ESM Fig. 5. Comparison of the fasting C-peptide/glucose characteristic of the ND individuals from the “first 100” and “next 150” from the INNODIA study: The plots show the scaled logarithm of fasted C-peptide/glucose ratio at each sampling point. Blue and red lines show the rapid and slow decline in beta cell function, respectively (based on the changes measured at the 2-year visit), and grey lines show the patients of intermediate category or without the 2-year C-peptide data.

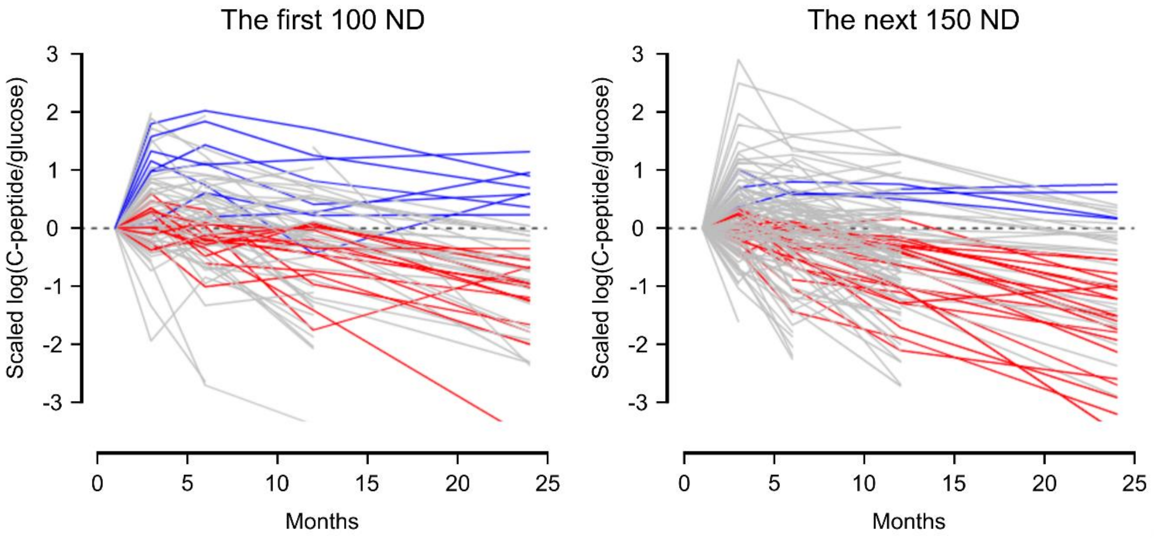

References

1. Bhosale SD, Moulder R, Kouvonen P, Lahesmaa R, Goodlett DR (2017) Mass Spectrometry-Based Serum Proteomics for Biomarker Discovery and Validation. Methods Mol Biol 1619:451–466. https://doi.org/10.1007/978-1-4939-7057-5_31 [doi]
2. Bhosale SD, Moulder R, Venalainen MS, et al (2018) Serum Proteomic Profiling to Identify Biomarkers of Premature Carotid Atherosclerosis. Sci Rep 8(1):9209. https://doi.org/10.1038/s41598-018-27265-9 [doi]
3. MacLean B, Tomazela DM, Shulman N, et al (2010) Skyline: an open source document editor for creating and analyzing targeted proteomics experiments. Bioinformatics 26(7):966–968. https://doi.org/10.1093/bioinformatics/btq054 [doi]
4. Moulder R, Välikangas T, Hirvonen MK, et al (2023) Targeted serum proteomics of longitudinal samples from newly diagnosed youth with type 1 diabetes distinguishes markers of disease and C-peptide trajectory. Diabetologia 66(11):1983–1996. https://doi.org/10.1007/s00125-023-05974-9
